# Barriers and Facilitators Experienced During the Implementation of Web-Based Teleradiology System in Public Hospitals of the Northwest Ethiopia: An Interpretive Description Study

**DOI:** 10.1155/2024/5578056

**Published:** 2024-06-06

**Authors:** Araya Mesfin Nigatu, Tesfahun Melese Yilma, Lemma Derseh Gezie, Yonathan Gebrewold, Monika Knudsen Gullslett, Shegaw Anagaw Mengiste, Binyam Tilahun

**Affiliations:** ^1^ Department of Health Informatics Institute of Public Health University of Gondar, Gondar, Ethiopia; ^2^ Department of Epidemiology and Biostatistics Institute of Public Health University of Gondar, Gondar, Ethiopia; ^3^ Department of Radiology School of Medicine University of Gondar, Gondar, Ethiopia; ^4^ Norwegian Centre for Ehealthresearch University Hospital of North Norway, Tromsø, Norway; ^5^ Management Information Systems University of South-Eastern Norway, Drammen, Norway

**Keywords:** barriers, facilitators, implementation, public hospitals, teleradiology, web-based

## Abstract

**Introduction:** Teleradiology allows distant facilities to electronically transmit images for interpretation, thereby bridging the radiology service gap between urban and rural areas. The technology improves healthcare quality, treatment options, and diagnostic accuracy. However, in low resource settings like Ethiopia, teleradiology services are limited, posing challenges for implementation. Therefore, this study is aimed at exploring the factors that facilitated or hindered the implementation of web-based teleradiology in the public hospitals of the South Gondar Zone, Northwest Ethiopia.

**Methods:** In this study, a purposive sampling method was employed to select seventeen participants, including hospital managers, physicians, emergency surgeons, and radiologists, for an in-depth interview (IDI). The interviews were conducted from March to May 2023. A reflexive thematic analysis was conducted using an abductive coding technique at the semantic/explicit level. Data were collected through semistructured interviews conducted face-to-face and virtually, with audio recordings transcribed, translated, and analyzed using Open Code version 4.02 software. Trustworthiness was ensured through prolonged engagement, reflective journaling, and review by coauthors.

**Results:** The study examined eight main themes, with barriers to sustainable teleradiology implementation falling into five categories: technological, organizational, environmental, individual, and workflow and communication. Conversely, identified facilitators included improved radiology service efficiency, system accessibility, collaboration opportunities, and user trust in the radiology ecosystem. Within each theme, factors with potential impacts on teleradiology system sustainability were identified, such as the lack of system handover mechanisms, absence of a central image consultation center, and inadequate staffing of full-time radiologists and technical personnel.

**Conclusions:** The study highlights the positive user perception of a web-based teleradiology system's user-friendliness and efficiency. Overcoming challenges and leveraging facilitators are crucial for optimizing teleradiology and improving service delivery and patient outcomes. A centralized consultation center with dedicated radiologists and technical personnel is recommended for maximizing efficiency.

## 1. Introduction

Teleradiology, the most advanced and rapidly evolving sector in telemedicine, involves the electronic transmission of medical images from one location to another for image interpretation and/or consultation [1]. Such service enhances patient care delivery by enabling off-site image interpretation and facilitating global collaboration with healthcare facilities [2, 3]. The utilization of teleradiology is indicative of the evolving landscape of clinical practice, service provision, and technological advancements [4]. The transition from analog to digital imaging techniques is the primary driver of teleradiology's explosive growth [5], which reflects how clinical practice and service delivery are evolving globally [4].

Teleradiology is widely adopted in the WHO European region, with 84% of member states utilizing it [6]. In Africa, among the few member states that implemented telemedicine, teleradiology accounts for 66% of the services [7]. The expansion of teleradiology can be attributed to several factors, including the demand for subspecialty reporting, rising emergency department visits, the need for prompt final reads, increasing acceptance and demand, and handling local overflow cases [5, 8, 9]. The promising growth of teleradiology in developed countries can be attributed to the expansion of Information Communication Technology (ICT) infrastructure [10], Picture Archiving and Communication System (PACS) [8], high-bandwidth Internet connections, collaboration with hospital leadership, and engagement with healthcare personnel [11].

However, in Africa, the utilization of teleradiology services varied among hospitals [11], resulting in fragmented implementation across diverse settings on the continent [8]. The adoption of telemedicine has been hindered by a combination of high upfront costs, insufficient infrastructure and connectivity, competing healthcare priorities, technological limitations, regulatory uncertainty, resistance from clinicians and IT staff, lack of political support, and concerns over data privacy and security [8, 9, 12–15]. In rural hospitals of developing countries with a shortage of radiologists, in-person referral consultations having to travel long distances are common. Limited access to radiology services due to long travel distances exacerbates delays, increases costs, and hampers timely diagnosis and treatment [16, 17]. Moreover, in the physical referral consultation approach, obtaining comprehensive patient information from referring clinicians poses challenges for consulting radiologists [18]. To address the challenges related to accessibility in resource-constrained countries, implementing contextually relevant teleradiology is highly recommended [19–22]. It improves patient care through remote image interpretation and collaboration with global healthcare facilities [2, 3], reflecting the changing landscape of clinical practice and digital imaging advancements [4, 5]. However, end users' intention to use was influenced by individual, environmental, and technological factors [23]. The acceptance and intention to adopt teleradiology soon were relatively low, with rates of 41.2% and 35.3%, respectively. Additionally, a significant portion (25%) of users did not utilize or plan to use teleradiology [24].

The Ethiopian federal government has set forth an ambitious vision to achieve a “Digital Ethiopia” by 2025, aligning with the WHO recommendation on digital health implementation strategy for the period of 2020–2025 [25]. To guide this transformative initiative, a digital health blueprint document was meticulously formulated and officially introduced in August 2021 [26]. Teleradiology was identified in the guiding document as a solution to enhance remote healthcare services in public hospitals within a short-term period. However, the government failed to implement teleradiology as planned, and challenges for implementation specific to the Ethiopian context remain unexplored. To bridge this gap, the authors developed and implemented a web-based teleradiology system in Amhara regional state public hospitals aimed to answer the following main research question:

What are the barriers and facilitators impacting end-user acceptance and system sustainability in the implementation of a web-based teleradiology system in Amhara Regional State public hospitals?

Examining this research inquiry holds significant importance as it offers valuable perspectives for various stakeholders. Firstly, it assists upcoming researchers in tackling unexplored obstacles like affordability. Secondly, it aids policymakers in shaping policies regarding system ownership and transfer. Thirdly, it helps administrative bodies in devising strategies and making well-informed decisions regarding organizational challenges. Lastly, it enables clinical practitioners to comprehend the benefits of the system for their medical practice.

## 2. Materials and Methods

### 2.1. Qualitative Approach and Research Paradigm

Our study utilized interpretive description as a methodology to explore the significance and contextual aspects of a particular phenomenon [27, 28]. This methodology adopts a structured approach that guarantees logical coherence, practice-oriented findings, awareness of biases, and consideration of practical implications when investigating individuals' perspectives [29, 30]. The study considered a pragmatic, theoretical, philosophical approach that prioritizes practical knowledge for decision-making and improved field practice, considering the diverse contexts involved [31]. This philosophical approach enables researchers to examine barriers and facilitators, emphasizing actionable insights to enhance end user acceptability, ensure system sustainability during implementation, and consider unique contextual factors and the importance of the research question [32, 33].

### 2.2. Researcher Characteristics and Reflexivity

The interviewer, who had an IT background, played an active role in various aspects of developing the web-based teleradiology system, including collecting requirements, participating in development and implementation, providing onsite training and support, developing user manuals, and offering virtual assistance. This involvement and the interviewer's technical expertise may have influenced the data collection and interpretation of findings.

To mitigate potential bias, the researchers implemented reflexivity by recognizing and acknowledging the interviewer's positionality, preconceived ideas, and insider perspective. The researcher engaged in self-reflection, sought feedback, and documented biases to promote reflexivity. Additionally, participants were provided information about the interviewer's background to uphold transparency.

During data analysis, the researchers employed strategies to reduce biases, such as incorporating multiple coders, seeking consensus, and utilizing iterative processes. The researchers also recognized the limitations and their impact on the study's validity and generalizability. Overall, the purpose of these reflexivity considerations was to enhance transparency, mitigate biases, and better understand the interviewer's influence on the findings.

### 2.3. Study Design and Study Period

The study employed an interpretative description study design. The study was conducted in South Gondar Zone public hospitals in Northwest Ethiopia from March to May 2023.

### 2.4. Study Setting

The study was conducted in public hospitals of South Gondar Zone, Northwest Ethiopia. According to the 2022/2023 Amhara Regional Health Bureau report, the zone encompasses 15 districts and 411 kebeles (the smallest administrative unit). The healthcare infrastructure in the area includes 10 public hospitals, 93 health centers, and 405 health posts, serving roughly 2.7 million populations. The clinical and medical imaging services in the zone public hospitals were provided by 21 specialists, 133 general practitioners, 26 emergency surgeons, and 27 medical imaging professionals (2 radiologists, 2 radiotechnologists, and 23 radiographers/imaging technicians).

### 2.5. Sampling Strategy

The study employed the maximum variation sampling method for participant recruitment to conduct in-depth interviews (IDIs). The purpose of employing a maximum variation sampling technique is to select a sample that is often more [34], facilitating a comprehensive understanding of the barriers and facilitators for sustainable web-based teleradiology implementation. Participants for the IDIs were purposively selected from hospitals where web-based teleradiology was implemented.

### 2.6. Description of Sample

The IDIs included healthcare professionals, such as physicians, radiologists, emergency surgeons, and hospital administrators, who were directly involved in the implementation process. Participants were selected from the referring and referral hospitals based on two criteria: they had not sent any image for consultation, and they actively participated by sending a larger number of images. This selection is aimed at gaining a comprehensive understanding of the challenges and facilitators encountered during the web-based teleradiology implementation.

### 2.7. Sample Size

The number of participants interviewed was determined based on the information power. According to the concept of information power, a larger amount of relevant information within the sample reduces the need for a larger number of participants in the study [35]. As a result, the study included 17 participants for the IDIs, and their informed consent was obtained.

### 2.8. Data Collection

A pilot-tested semistructured interview guide was utilized to collect the data. Initially, it was prepared in English and later translated into Amharic, the local language of the study area. To ensure the relevance and coverage of our research questions, we consulted with experts on our team. The final survey, which aimed to explore barriers and facilitators of sustainable web-based teleradiology implementation, consisted of open-ended questions. These questions allowed respondents to express their experiences and insights freely. Additionally, probe questions were used during the interviews to gather detailed information. A comprehensive interview semistructured guide was developed based on the Technology-Organization-Environment (TOE) framework [36, 37]. The principal investigator (AM) created the interview guide to gather participants' views on the challenges, facilitators, and sustainability of web-based teleradiology implementation. Field notes were taken to capture contextual details, and the average interview duration was 55 min.

### 2.9. Methods of Approach and Settings of Data Collection

The principal investigator (AM) and a research assistant (AT) from the Department of Health Informatics approached the study participants for face-to-face and virtual interviews via Google Meet (free service provided by Google), a video conferencing tool. The participants were given the freedom to choose the time and location of the interviews. For face-to-face interviews, the researchers visited the participants' offices at their health facilities, ensuring a calm, secure, and comfortable environment with minimal disruptions. This approach is aimed at maintaining the quality of the recordings and encouraging open discussions. The principal investigator (AM) and research assistant possessed extensive expertise in collecting and analyzing qualitative research data. Only the researchers and the selected participants were involved in the study, with no other individuals participating.

### 2.10. Data Coding Process

The data processing procedures included audio verbatim Amharic transcription and English translation, conducted by two researchers (AM and AT). The transcribed file underwent anonymization/deidentification and was imported into Open Code 4.02 software for analysis. Experienced researchers (AM and AT) independently performed data coding after multiple reviews of the transcribed texts. Both researchers were trained and experienced in qualitative data analysis methods and software utilization.

### 2.11. Data Saturation

In the current study, the term data saturation refers to the data collection point where new interviews provide minimal or no additional information relevant to the research question. Based on previous literature, it is suggested that a minimum of 12 interviews is needed to reach saturation [38].

### 2.12. Data Analysis

Recorded interviews and field notes were transcribed verbatim, initially in Amharic and later translated into English. Data analysis was conducted using a reflexive thematic analysis approach developed by Braun et al. [39]. This method was chosen for its adaptability and theoretical flexibility in qualitative health research [40]. This approach's main objective is to fully understand participants' experiences, perceptions, and comprehension of a specific phenomenon (web-based teleradiology implementation). Author AM was responsible for the initial data analyses, which were imported into Open Code 4.02 software, streamlining the coding, data analysis, and organization processes and enhancing efficiency and accuracy. Line-by-line coding was utilized to identify relevant patterns, with the resulting codes representing emerging patterns, concepts, or ideas and facilitating the manageable analysis of the data.

To ensure the coherence and plausibility of the identified themes in answering the research questions, all authors met during step four and subsequent stages of the reflexive thematic analysis. This study employed an abductive coding approach [41]. The study findings were reported following the guidelines of the Standards for Reporting Qualitative Research (SRQR).

### 2.13. Trustworthiness

The study ensured credibility through expert review and pilot testing of the interview guide, involving a diverse range of participants and providing documented evidence. Furthermore, member checking was employed by providing participants to review and validate the interpretation. Experienced researchers (AM and the research assistant AT) engaged with participants using clear and straightforward Amharic language, ensuring that participants could express their feelings without any difficulties related to language comprehension. The research team had a prolonged engagement with participants. The study further established confirmability by employing collaborative coding and adhering to qualitative research standards, while dependability was ensured through recorded coding and review processes. The study included the critical reflexivity including the data collector's role, bias, and potential influence on the research process and findings. The study enabled transferability by providing a thick description of documentation of research methods including data collection and analysis process, research context, study setting, and participant demographics. Finally, to ensure accuracy, we presented our findings in accordance with the SRQR guideline.

## 3. Results

### 3.1. Background Information of the Study Participants

Table 1 describes the overview of the study participants' characteristics. Seventeen IDIs were conducted for this study, involving hospital managers, medical doctors, emergency surgeons, and radiologists. Out of the total participants, 15 were males, and two were females. The median age of the participants was 30 years. All IDI participants were actively engaged in the healthcare delivery system.

**Figure 1:**
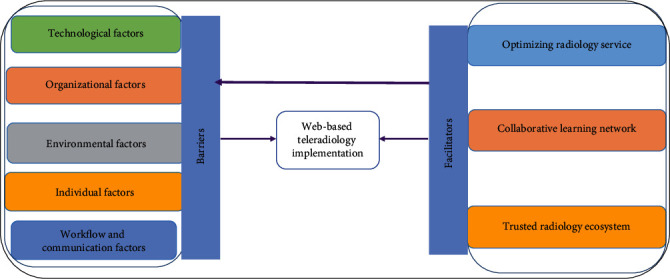
Visual framework for barriers and facilitators of sustainable web-based teleradiology implementation, Northwest Ethiopia.

This study extensively explored the factors that contribute to or impede the successful implementation of web-based teleradiology systems. The categorized findings, represented in Figure 1, highlighted technological, organizational, environmental, individual, and workflow and communication factors. On the other hand, improved radiology service efficiency, enhanced accessibility and collaboration opportunities, and the establishment of a trusted radiology ecosystem were considered as facilitators for the implementation.

### 3.2. Potential Barriers to Implementation

Although digital health holds significant promise for improving the healthcare service delivery, there are potential barriers that can hinder its implementation. The study identified five subthemes that shed light on how these barriers can hinder the initiation and implementation of web-based teleradiology. These subthemes include technological factors, organizational factors, environmental factors, individual factors, and workflow and communication factors.

#### 3.2.1. Theme 1: Technological Factors

Participants mentioned that technological challenges related to ICT infrastructure and communication impeded the successful implementation of web-based teleradiology. These challenges encompassed unreliable Internet connection, unstable local area network, server downtime, and the lack of notifications. Consequently, healthcare access was delayed, and the quality of patient service was compromised. Addressing these issues is crucial for the efficient functioning of the teleradiology system.

Expanding internet service to our remaining service areas poses a significant challenge due to the small internet bandwidth. As a solution, we have centralized the internet access in one room only and assigned doctors to hand the image upload and result dispatch the PDF radiology report when ready to the consulting physician via our Telegram channel. (IDI-08, hospital manager)

The respondents also underlined the significance of the notification feature in the web-based system, emphasizing its favorable influence on the prompt delivery of clinical services and its role in streamlining professionals' workflow.

… though the current radiology turnaround time is promising, adding a notification feature to the system could further improve it significantly. (IDI-16, radiologist)

The IDI participants' observations revealed significant implications regarding technical challenges in the implementation of sustainable web-based teleradiology. These challenges, including the absence of technical experts in the field and the lack of timely onsite support services, posed obstacles to the smooth operation of the system. Addressing these challenges is important to a smooth-running teleradiology system implementation.

Our institution faced a one-month internet connection suspension caused by a technical issue. Despite the efforts of district experts, the problem persisted. However, after one month and 15 days, remote support from federal experts and onsite assistance from telecommunication professionals successfully resolved the issue. (IDI-02, hospital manager)

Another participant also highlighted that

Teleradiology technology is highly favored when there is reliable network access and a stable and fast Internet connection. (IDI-16, radiologist)

#### 3.2.2. Theme 2: Organizational Factors

Participants explained that successful implementation and sustainability of web-based teleradiology requires addressing inadequate funding and unreliable power. Limited budgets hinder investments in infrastructure, equipment, and skilled personnel, limiting scalability and service quality. Overcoming these challenges is crucial for seamless and efficient teleradiology services.

In my opinion, despite the current free access to teleradiology services, guaranteeing its sustainability requires adequate financial support. Hence, collaborating with non-governmental organizations to acquire additional funding emerges as a vital solution. These funds will allow us to incentivize radiologists and scale up our limited IT infrastructure, thereby securing the service's future. (IDI-07, medical director)

Another participant also explained.

Institutional barriers in teleradiology implementation primarily arise from budget constraints and system implementation. Hospitals frequently claim inadequate funds as a reason for not being able to enhance network and internet services. (ID-17, radiologist)

Participants highlighted that the shortage of technical personnel and radiologists, as well as staff turnover, poses significant challenges to the field. These issues disrupt the workflow, hinder system implementation, and cause delays in diagnosis and patient care. Addressing these challenges is crucial for efficient and high-quality healthcare delivery.

Currently, there are no technical professionals available to repair the machine when we encounter X-ray machine failure. As a solution, we are trying to persuade technicians from the machine provider's side by covering all the expenses. However, this approach might not be sustained because of its feasibility and sustainability. (IDI-09, hospital manager)

A participant emphasized the impact of the shortage of radiologists on service delivery:

The shortage of radiology professionals is impeding the prompt delivery of services, making it necessary to adopt teleradiology as a means to enhance access to medical treatment. (IDI-17, radiologist)

Participants underlined that insufficient incentives, weak administrative support, and a lack of organizational commitment affected the successful implementation of the system by suppressing motivation and limiting resource allocation. Overcoming these obstacles is crucial for effective implementation.

Though the current teleradiology service is free, its sustainability is uncertain. In my opinion, to sustain the system, considering collaboration with non-governmental organizations to secure future funding, provide incentives for radiologists and establish system monitoring and evaluation processes is very important. (IDI-07, medical doctor)

#### 3.2.3. Theme 3: Environmental Factors

Participants explained that insufficient regulatory guidelines and a lack of performance indicators specific to teleradiology pose challenges to its sustainable implementation. The absence of clear regulations can lead to ambiguity, thereby impeding system development.

The main obstacle to accepting the web-based teleradiology system is not the concept of teleradiology itself but rather the absence of guidelines and a user-friendly environment that facilitates the seamless utilization of the software. (IDI-10, medical director)

The interviewees remarked that the lack of a responsible body and absence of institutionalization, coordination, and efficiency due to unclear system ownership or handover processes were impacting the long-term sustainability of the system implementation. Addressing these crucial issues is paramount to building a solid foundation for effective system implementation and operation.

To ensure the system's sustainability, I suggest the Ministry of Health/Regional Health Bureau establish an independent consultation center or a separate organization or institution, like a university, to take ownership and responsibility and provide continuous support for the initiative, considering it as a cornerstone project. (IDI-16, radiologist)

Moreover, another participant also emphasized:

Sporadic delays in radiology turnaround times may arise due to workload and lack of personal ownership. However, technology transfer to other institutions and actively promoting its advantages holds significant potential for the long-term sustainability of the system. (IDI-17, radiologists)

According to the participant's observation, teleradiology implementation requires the active participation of a broad range of stakeholders with diverse expertise since maximizing effectiveness requires collective contribution from all stakeholders rather than relying solely on specific individuals. However, failure to do so would lead to limited knowledge, resistance, misalignment with existing systems, and poor adoption. To ensure that the project achieves its full potential and benefits all involved, actively engaging stakeholders is paramount.

Active support and coordination among stakeholders like Ethio-Telecom, hospital administration, and the mayor's office, and collaboration with colleges and universities for capacity building are necessary for technology implementation. (IDI-13, hospital manager)

Other participants also highlighted that

… although the regional health bureau leads the charge in bringing teleradiology to life, its sustained existence relies solely on the budgetary capabilities of individual hospitals. (IDI-17, radiologist)

Participants highlighted the lack of assigned radiologists as a major hindrance to web-based teleradiology implementation. This deficiency negatively impacts effective communication and collaboration among healthcare professionals, resulting in delayed diagnoses and compromised patient care coordination. It also disrupts workflow, leading to longer turnaround times and reduced efficiency. It is crucial to establish a dedicated consultation center, implement robust communication platforms, and establish clear protocols for radiologist assignment and availability to address challenges.

In my opinion, to enhance the sustainability of the teleradiology system, it is good to assign a full-time radiologist and establish an independent radiology consultation center. This approach ensures consistent and timely service, resolves delays in radiology report submission, and promotes accountability. (IDI-15, medical doctor)

In addition, another interviewee also explained the major challenge associated with the lack of assigned full-time radiologists and the lack of a radiology image consultation center.

The absence of a full-time radiologist and an independent radiology consultation center can lead to delays caused by human factors rather than technological issues. It was suggested that specialized hospitals could assume the responsibility to address this issue. (IDI-12, emergency surgeon)

#### 3.2.4. Theme 4: Individual Factors

According to the interviewees' observations, challenges such as low professional readiness, lack of familiarity, technical skill gaps, and limited knowledge significantly hinder the successful implementation of teleradiology. The unfamiliarity with the system has prevented its full potential utilization as intended, resulting in suboptimal utilization and resistance towards adopting the technology.

Initially, my knowledge of web-based teleradiology was limited to basic information, and I was not eager to use the system. However, upon integrating it into my practice, I swiftly realized its significant advantages and contributions to my work. (IDI-05, emergency surgeon)

Moreover, they also added that without end users' commitment, it is impractical to think about the system's implementation and sustainability. To overcome these challenges, participants commented that organizations need to actively address user concerns and cultivate a culture that embraces technological advancements and welcomes positive change.

I noticed some clinicians exhibit resistance to adopting teleradiology and I assumed this resistance stems from a lack of understanding regarding the importance of participating in teleradiology. (IDI-09, hospital manager)

Another participant also commented that the implementation challenge is multidimensional.

… I believe that the challenge of utilizing a teleradiology system is not necessarily linked to the system itself but rather to the professional's motivation to utilize it and the availability of network connectivity. (IDI-10, medical director)

#### 3.2.5. Theme 5: Workflow and Communication Factors

Participants identified delayed consultation processes and late radiology report submissions as factors that impact radiology workflow and communication. If not effectively managed, these delays can be attributed to the referring and referral hospitals, leading to heightened patient anxiety, as well as delays in diagnosis and treatment.

The delay in radiological results is influenced by both the referring physicians when they fail to provide the necessary information accurately and the consulted radiologists when they are excessively overloaded, which altogether leads to a delay in the turnaround time of the radiology report. (IDI-10, medical doctor)

Similarly, another interviewee also complemented how the radiologist's workload was highly attributed to the delay in turnaround time.

… there was a time when an image sent in the morning was not commented on and returned until the evening, and there was a time when an image was overlooked entirely due to excessive workload. (IDI-17, radiologist)

It was highlighted that the technical challenges associated with system integration resulted in the need for recaptured images. These challenges, in turn, adversely impact the accuracy of diagnoses, patient care, and workflow efficiency.

In most of our cases, uploading the digital images directly from the computer was not practical due to unresolved technical issues … unfortunately; this approach compromises the image quality since it depends on the quality of the camera, potentially impacting the accuracy of our radiology report due to the impracticability of precise measurements. (IDI-07, medical doctor)

### 3.3. Facilitators for Web-Based Teleradiology System Implementation

The current technological era presents a significant opportunity for implementing and enhancing radiology service provision. There is a substantial demand to support healthcare services with digital technologies, including teleradiology. The study explores facilitators for successful web-based teleradiology implementation, which fall into three subthemes: improved radiology service efficiency, enhanced accessibility and collaboration opportunities, and establishment of a trusted radiology ecosystem.

#### 3.3.1. Theme 6: Improved Radiology Service Efficiency

Participants acknowledged the advantages of the implemented web-based teleradiology system. It empowered end users to enhance treatment quality through high-quality radiology reports and facilitated informed decision-making. As a result, it improved patient satisfaction by saving time and reducing unnecessary costs.

Teleradiology offers a multitude of benefits, most notably the ability to ensure high-quality treatment for patients based on the accurate report reviewed by the appropriate professionals. … significantly reduces the risk of misdiagnosis. In addition, since teleradiology eliminates the need for patients to travel to other locations for referrals physically, it saves time and unnecessary costs associated with traveling. (IDI-05, emergency surgeon)

Another interviewee emphasized the contribution of teleradiology in enhancing system efficiency:

Based on my observation, the teleradiology system is an invaluable tool for minimizing patient discomfort, as its comprehensibility and efficiency reduce instances of mistreatment and increase satisfaction with reduced transportation and expenses. I am genuinely delighted with the system, as it effectively shortens the time patients spend seeking image interpretation services elsewhere. (IDI-07, medical doctor)

Furthermore, the positive attitude of users towards the system encouraged its utilization. They emphasized that the user-friendly interface and easily understandable content had a positive impact on users' attitudes toward teleradiology implementation. This, in turn, enhanced service delivery and reduced waiting time for radiology reports.

I have not noticed any gaps in the system except for those that emanated from the users' side. In my opinion, this system is user-friendly and does not require extensive computer knowledge (a brief orientation is sufficient). Moreover, the contents are clear and easily understandable, which disproves health professionals' perceptions. (IDI-12, emergency surgeon)

Another interviewee also remarked on the contribution of the system from the perspective of waiting time reduction:

Following the implementation of the system, there has been a substantial improvement in the turnaround time, surprisingly, even within minutes. However, there were very rare times we encountered delays of up to two days. In general, I am very satisfied and motivated with the teleradiology system and I have a plan to use it in the future consistently. (IDI-09, hospital manager)

#### 3.3.2. Theme 7: Accessibility and Collaboration Opportunities

Participants reported that the implementation enhances accessibility, allowing professionals to collaborate on radiology cases from any location with Internet connectivity at any time. It eliminates the need for physical transfers, streamlines the process, and improves turnaround times. The system also provides a convenient solution through an audit trail and tracking system, enhancing the system.

The web-based nature of the teleradiology system opens up possibilities for utilization not only by local radiologists but also facilitates the engagement of international radiologists, enhancing its overall effectiveness and viability. Furthermore, its unrestricted accessibility, regardless of location or time constraints, enables radiologists to cover a wide area of patient distribution efficiently. (IDI-17, radiologist)

Moreover, participants indicated that since the implementation needs staff commitment and collaboration, the hospital manager's role accomplishment is very important in leading and coaching the staff.

Obviously, I have heard a proverb, “An institution mirrors its leader.” The quote suggests that strong leadership is crucial in driving exemplary work within an organization. Fortunately, we have encountered no issues with leadership commitment at both the institutional and departmental levels, despite the complexities that existed during the establishment period. (IDI-11, hospital manager)

Interviewees emphasized the advantages of the system's feedback section. They found it beneficial for sharing radiologists' insights, recommendations, and suggestions, enabling continuous learning and improvement. The platform facilitates seamless collaboration and consultation among radiologists and referring physicians, supporting efficient multidisciplinary discussions and decision-making, which helps to promote timely and comprehensive patient care, leading to optimal outcomes.

The feedback section plays a vital role, especially for remote clinicians, bridging knowledge gaps when effectively utilized. It provides essential information and recommendations to enhance our practice. Radiologists guide us to resend images for poor quality, additional views, or advanced medical imaging needs. Overall, the feedback section ensures the quality and effectiveness of our service provision. (IDI-12, emergency surgeon)

#### 3.3.3. Theme 8: Trusted Radiology Ecosystem

Interviewees highlighted the importance of simplifying standardized report submission and fostering user trust as crucial factors for the successful implementation of web-based teleradiology. They also emphasized that optimizing the reporting process improves efficiency and data quality while establishing trust in the technology promotes widespread adoption and utilization.

The web-based teleradiology system assists us in generating professionally standardized confidentiality reports that adhere to international standards. (IDI-16)

Furthermore, participants underscored the fundamental importance of establishing a strong foundation of trust in data security and patient confidentiality for the successful implementation of the system. They highlighted that users will fully embrace and utilize this transformative technology only when they feel confident that their data is protected and patient information remains private.

Confidentiality is paramount in our teleradiology system. Access to patient data is strictly limited to authorized users (physicians, radiologists, and system administrators) who utilize unique username and password credentials. Additionally, the system provides transparent documentation of every image consultation, explicitly identifying the responsible consulting clinicians and the radiologists who provided comments and submitted the report, including their names and professions. (IDI-0, medical doctor)

## 4. Discussion

This paper specifically addresses the barriers and enablers related to the implementation of sustainable web-based teleradiology. It recognizes the challenges that can impede successful implementation while also exploring the factors that can facilitate the process. The importance of web-based teleradiology lies in its ability to provide convenient access to patients' previous examinations, thereby enhancing accuracy and facilitating a comprehensive understanding of their medical history. IDIs with healthcare professionals were conducted to identify the barriers and facilitators associated with teleradiology implementation, resulting in a more comprehensive understanding and the web-based teleradiology. Despite the clinical benefits of teleradiology in a resource-limited setting, such as improved diagnostics and cost savings through reduced unnecessary treatments and medications [42], our study identified several potential obstacles in the implementation process. These barriers encompassed technological, organizational, environmental, individual, and workflow and communication factors. Conversely, the study also identified facilitators that contribute to the successful implementation of web-based teleradiology systems, such as enhanced radiology service efficiency, improved accessibility and collaboration opportunities, and the establishment of a trusted radiology ecosystem.

The technological factor associated with poor ICT infrastructure and delayed technical maintenance pose a remarkable challenge for the sustainable implementation of web-based teleradiology. Findings in the study are consistent with previous research studies indicating that poor ICT infrastructure hampers the widespread adoption of technology tools in healthcare, posing significant technical obstacles [10, 14, 43–46]. The possible explanation for this could be establishing a robust ICT infrastructure serves as a potential explanation, bridging the gap and unlocking the full potential of successful digital health initiative implementation in the healthcare industry [47–49]. The findings suggest that adequate funding for improving ICT infrastructure, timely technical support, and ongoing maintenance are essential for ensuring the successful implementation and long-term sustainability of web-based teleradiology systems in healthcare settings.

The current study revealed significant organizational challenges for teleradiology implementation, including unstable electricity, inadequate budget allocation, lack of technical personnel and radiologists, and lack of organizational commitment. This finding is consistent with a study conducted in Iran, highlighting the hindrances posed by inadequate infrastructure and high initial costs in establishing teleradiology services [14]. Furthermore, other similar studies demonstrated that identified organizational factors such as inadequate budget, low organizational readiness, and legal rules as significant impeding factors for successful implementation of web-based teleradiology systems [50–52]. The possible explanation could be high initial investment is needed to avail adequate ICT infrastructure, to rain end users, and to purchase equipment.

Poor implementation of teleradiology can be attributed to environmental factors like the absence of regulatory documents and inadequate stakeholder engagement, which aligns with previous studies [43, 53]. These studies emphasize the importance of clear communication strategies and engaging multiple stakeholders for successful teleradiology implementation. In our study, we found that the lack of system ownership and poor collaboration had an impact on the implementation, which is consistent with findings from studies conducted in Mali [11] and Berlin Trauma Hospital (Germany) [10]. These studies highlight the critical importance of ownership and collaboration involving national radiologists and healthcare personnel for the successful implementation of teleradiology. Similarly, the lack of specific regulatory guidelines and policies' impact on web-based teleradiology implementation is explained by the study participants of the current study. Previous studies [43] corroborate these findings, highlighting regulatory barriers that impede the development, implementation, and operation of digital health in developing countries. Ethiopia has developed several digital health documents, including policies, strategies, guidelines, roadmaps, architecture, plans, and a directive, despite the absence of a specific teleradiology implementation policy [54].

Our study highlights that individual-level factors such as low awareness, limited knowledge, and inadequate technical skills hinder end users from using the system. The findings of this study are consistent with previous studies demonstrating that inadequate skills [46]. Furthermore, additional studies have shown that positive user perceptions of the implemented technology [6, 55], as well as user involvement in the development of healthcare systems [56], are factors that contribute to successful system implementation and the growth of teleradiology. The possible rationale for this could be end users, including radiographers, radiologists, and referring clinicians, play a vital role in the effective operation of teleradiology systems [57]. However, unresolved issues such as limited comfort with technology and older age groups [36], as well as language disparity [58]continue to impede its implementation.

Positive user experiences and system efficiency were found as important factors in improving radiology service delivery and enhancing treatment quality, satisfaction, and informed decision-making. These findings align with previous research highlighting the potential of web teleradiology systems to improve radiology report quality, address radiologist shortages, and facilitate the sharing of specialist expertise and equipment in resource-limited hospitals [53, 59, 60]. These possible explanations could imply applying webpage teleradiology, and three significant challenges can be addressed simultaneously: enhancing the quality of radiology reports, overcoming the radiologist shortage, and promoting resource sharing among hospitals [13]. Moreover, this approach ensures positive user experiences and efficient systems, resulting in improved treatment outcomes, increased patient satisfaction, and enhanced decision-making capabilities.

Participants confirmed that the web-based system's accessibility and collaboration opportunities are significant enabler for the use of web-based teleradiology. This finding is consistent with previous studies indicating that the implementation of web-based teleradiology would help to promote the education of consulting clinicians [61].

The convenience of easy access anywhere and anytime, facilitated by an Internet connection, along with enhanced collaboration among professionals across countries, could justify this observation. In addition, interviewees highlighted the system's feedback section, enabling convenient consultation with radiologists and promoting collaboration. The study findings align with previous studies [53, 62], emphasizing the importance of radiologist feedback in clarifying diagnoses. Administrative bodies should consider the positive impact of the feedback section when expanding teleradiology to other public hospitals.

The trusted radiology ecosystem relies on convenient report preparation and submission, as well as user trust in system security and confidentiality, as a critical enabling contributor in facilitating the successful implementation of web-based teleradiology. The study on privacy and security in teleradiology highlighted the importance of meeting ethical and regulatory requirements [63]. Additionally, our research and previous studies emphasize that user-friendliness is a crucial factor for the success of digital technologies [64]. The possible explanation for this could be fulfilling ethical and regulatory requirements ensures privacy and security in teleradiology. At the same time, user-friendliness enhances the adoption and efficiency of digital technologies, providing a possible explanation for these findings.

### 4.1. Limitations of the Study

The limitations of this study should be acknowledged when analyzing its results. The perspectives of hospital managers, while contributing valuable insights, may not comprehensively represent the sentiments of the entire healthcare professional population as their involvement was limited only to the administrative aspects. Consequently, interpreting the findings with caution is recommended. Furthermore, the present investigation fails to include the perspective of patients. Future studies would benefit from the inclusion of both patient and provider viewpoints to facilitate a comprehensive understanding of the user experience.

### 4.2. Implications for Practice

The findings of this study provide guidance for the future implementation of teleradiology in public hospitals, specifically focusing on optimizing medical imaging services for rural populations. To achieve this goal, it is important to design a user-friendly web-based teleradiology system that takes into account potential barriers. By doing so, it can complement existing practices and make the most of limited resources. However, for a sustainable and effective implementation of a web-based teleradiology implementation, certain factors need to be addressed. These include providing technical support for end users, improving ICT infrastructures, and managing issues related to unstable electricity. In addition, it is crucial to develop regulatory documents that facilitate the transfer of implemented and tested home-grown digital health technology solutions, while also designing a strategy to monitor their practicality. Furthermore, environmental factors must be taken into consideration prior to implementation. This should include establishing consultation centers and assigning independent full-time radiologists and technical personnel to sustain the implementation.

## 5. Conclusions and Recommendations

The findings of the study highlighted the acceptability of the piloted web-based teleradiology system, though, the successful implementation of this system faces various challenges related to technology, organization, environment, individuals, and workflow and communication. On a positive side, the study identified three key themes as facilitators, namely, improved radiology service efficiency, accessibility and collaboration opportunities, and a trusted radiology ecosystem which significantly contributed to the successful implementation of web-based teleradiology. Optimizing web-based teleradiology, improving service delivery, and achieving better patient outcomes require addressing barriers and leveraging facilitators. Establishing a centralized consultation center with full-time assigned radiologists and technical personnel and integrating the web-based teleradiology with PACS is recommended to optimize resource utilization, foster collaboration, and facilitate the integration of services for effective large-scale implementation of teleradiology.

## Figures and Tables

**Table 1:** Participants' characteristics.

**Code**	**Age**	**Sex**	**Profession**	**Current administrative position**	**Experience (in a year)**
IDI-01	27	M	Medical doctor	Medical director	4
IDI-02	33	M	Health officer	Hospital manager	7
IDI-03	27	M	Medical doctor	Medical director	2
IDI-04	40	M	Medical doctor	No	4
IDI-05	29	F	Emergency surgeon	No	2
IDI-06	32	M	BSc in nursing	Hospital manager	9
IDI-07	26	M	Medical doctor	No	2
IDI-08	33	M	Public health	Hospital manager	10
IDI-09	33	M	Nurse	Hospital manager	11
IDI-10	27	M	Medical doctor	Medical director	2
IDI-11	28	M	Health officer	Hospital manager	6
IDI-12	30	M	Emergency surgeon	No	7
IDI-13	34	M	Public health	Hospital manager	9
IDI-14	26	F	Medical doctor	No	2
IDI-15	27	M	Medical doctor	No	2
IDI-16	33	M	Radiologist	No	3
IDI-17	32	M	Radiologist	No	4

Abbreviation: IDI = in-depth interview.

## Data Availability

All data generated or analyzed during this study are included in this manuscript.

## Nomenclature

CD: compact disk
CT: computed tomography
FMOH: Federal Ministry of Health
HSTP: Health Sector Transformation Plan
ICT: Information Communication Technology
LMICs: lower- and middle-income counties
mHealth: mobile health
MRI: magnetic resonance imaging
PACS: Picture Archiving and Communication System
SSA: sub-Saharan Africa
WHO: World Health Organization
